# Typhoid Fever

**Published:** 1904-10

**Authors:** 


### SELECTIONS AND ABSTRACTS.

### TYPHOID FEVER.*

Gentlemen of the Huntingdon County Medical Society: To attempt to write a paper on typhoid fever, a disease so common and familiar to every one, requires no little courage. You are all acquainted with its history, etiology, pathology, diagnosis and treatment. I imagine some of you are thinking, "Then why inflict us with a paper on a subject with which we are already acquainted?" I do not propose to lead you over old and familiar routes, but to ask you to follow me into some of the by-ways and some of the newly explored regions. Although we may not find better tools for our thereapeutic "armamentarium," we may get in a position where we may enjoy a broader view of the subject.

We recogonize that the bacillus of Eberth is the cause of typhoid fever, and that the symptoms are due to the absorption of the toxic product of that organism.

Linsly and Stone, in the *Medical Record,* say that the bacillus cóli commúnis is an etiological factor in may diseases, and that these cases of pseudotyphoid enteritis produced by drinking water from wells contaminated by sewage infiltration do not give the Widal reaction. Cases are on record where milk cans washed in water from wells the water from which contained the bacillus cóli commúnis, so infected the milk that persons using it became sick with enteritis or pseudotyphoid fever. In many cases of typhoid fever a double infection exists—the primary infection from the bacillus cóli commûnis and the secondary infection from the typhoid bacillus. In about twenty-five per cent. of cases of typhoid fever you will find the bacillus typhosus in the urine. In an editorial in the Journal of the American Medical Association for March 9, 1901, you will find some very interesting and important facts set forth. He says that the presence of typhoid bacilli in the urine

*Read by Dr. R. Myers, M.D., of Huntingdon, before the Huntingdon County Medical Society, September 8, 1903.

of a patient ill with enteric fever is important not only to the patient and his friends, but the entire community. It is important to the patient because it may mean cystitis, pyelitis and a prolonged and imperfect restoration.

Newfield, in one of the German journals of December 20, 1900, has presented this aspect of the case: He says that the danger from infected urine is greater than that from feces similarly infected because a given quantity of urine will contain, as a rule, a larger number of typhoid germs than will a similar quantity of feces. In one of Newfield's cases he estimated the number of bacilli at 100,000,000 per cubic centimeter. An article of clothing, a sheet, a bed-pan, a urinal, the hands or genitals may be soiled with urine and not be noticed as you would notice had they been soiled with fecal matter. Guyn had a case where he found typhoid fever bacilli in the urine five years after the attack of typhoid. Thus a patient may unwittingly be the cause of infection and start an epidemic of the disease. Dr. Eugene Wasdin, of Buffalo, N. Y., in a paper published in the *Therapeutic Gazette*, May 15, 1902, made the statement that the normal cycle of an uncomplicated case of typhoid infection is fourteen days, and that the primary germ colony in this disease can not be considered as taking place in the intestinal canal, but that the weight of evidence shows it to be normally an invasion of the respiratory tract; that the bacillus of Eberth invades the general circulation from this primary colony and from the blood current it may give rise to all of the well-known terminal expressions, such as the infection of the intestinal canal, the gall bladder, the urinary bladder, the serous membranes, spleen, bone marrow, etc. He further states that when a case continues to twenty-one days, the fact is explainable on the hypothesis that the primary intoxication of fourteen days is overlapped by the secondary toxic influence from the bowel, which must commence about the end of the first week, thus making the acme of the fever the time of the greatest intoxication, in these complicated cases.

Now for another view of the subject: Birt reports the results of inoculation with anti-typhoid serum in an epidemic of typhoid fever which occurred in South Africa from September, 1900, to September, 1901. During this period there were one thousand two hundred and ten cases. Nine hundred and forty-seven cases were not inoculated, and the mortality of those cases was fourteen and twenty-five one-hundredths per cent. On the other hand, two hundred and sixty-three cases had been inoculated with typhoid vaccine, six to eight months previously, and the mortality in these was only six and eight-tenths per cent. At Maidstone Asylum, Parry reports one hundred and five of the attendants were not inoculated and nineteen had typhoid. Ninety-three were inoculated and among these there was not a single case. In one of the Red Cross hospitals all of the nurses who had been inoculated escaped while nursing patients during a severe epidemic of typhoid. One nurse refused to be inoculafed, and she was the only one who contracted the disease. He also mentions that of ninety-two patients admitted at Kroonstadt, fifteen had been inoculated and seventy-seven had not been inoculated. Of these eleven patients died, one that had been inoculated once and ten that had not been inoculated. Of two thousand six hundred and sixty-nine uninoculated and seven hundred and twenty inoculated soldiers in Egypt during 1900, sixty-eight of the former died and only one of the latter died. I might present more statistics on this line, but the above-mentioned are sufficient to show the advantage of inoculation in fortifying against typhoid fever.

The complications of typhoid fever are quite numerous, and have manifested themselves in every portion of the body. McClintock notes that he has found nineteen cases of meningitis complicating typhoid fever, in all of which the bacillus was present. The list of complications, in which typhoid bacilli have been found, is long. A few of them are abscess of the brain, of the thyroid gland, and of the liver. In studying the pathology of these complications we come in contact with the dynamics of the human organism. The bacillus typhosus with its toxins as the invading force and the anti-bodies, as they are called by Wasdin, or the alexin of the phagocytes, the defensive forces. From the primary point the bacilli enter the blood, where they are drawn together and held by the agglutinin. This is often drawn away and the bacilli form nuclei, from which we have these abscesses. Matalinkoff says that the conditions seem too complex and too little understood to permit of any more definite explanation. In studying the newer lines of treatment, I found quite a lengthy paper in the *Therapeutic Gazette*, by our old friend, Dr. H. G. McCormick, of Williamsport, in which he reports that he has treated four hundred and eight cases, of which twenty-two have died; thus having a mortality of only five and four-tenths per cent. His treatment, as you all know, is guaiacol, in two minim doses, every two hours, after having moved the bowels with calomel. The guaiacol is given entirely for its antiseptic effect upon the intestines. To reduce the temperature he has the right illiac region thoroughly cleansed, and then slowly drops from five to ten drops of guaiacol on the surface and then rubs it in thoroughly. The part is then covered with oiled silk or waxed paper. The most interesting portion of the doctor's paper is that in which he gives his experience in the use of the acid sulphate of soda. After an interesting experience with guinea pigs, demonstrating the effect of the acid sulphate of soda on the typhoid bacilli and its power to increase phagocytosis, he treated four cases and then gave the following conclusions:

First, that the acid sulphate of soda is germicidal to typhoid bacilli in a solution of 1 to 300.

Second, that it increases leucocytosis.

Third, that it neutralizes typhotoxin.

He gives of a solution of 15 grains acid sulphate of soda to 4 ounces of water from fifteen to twenty minims every two hours.

The treatment which interests me most is the acetozone treatment. Dr. Wasdin, of Buffalo, treated twenty-seven cases. In these cases there were no complications and no deaths. In a large number of them the duration of the disease was reduced to the fourteen-day cycle. His treatment was as follows: The bowels were thoroughly moved by one-grain doses of calomel combined with one grain of aloin and two grains of guaiacol carbonate. This dose was repeated every four hours, until the canal was well flushed. The patient was given fifteen hundred to two thousand cubic centimeters of twenty-four-hour-old solution of acetozone daily. In his conclusion he says that the special application of acetozone in typhoid fever enables us to obviate intestinal infection and absorptive toxemia therefrom, thus favoring the formation of protective anti-bodies and limiting in many cases the disease to its normal cycle.

Dr. Frederick Harris, of the Cook County Hospital, Chicago, Ill., treated one hundred and twenty-eight cases with the following results: The average stay of the one hundred and twenty-eight patients in the hospital was thirty-one and four one-hundredths days. Of the one hundred and twenty-eight patients, one hundred and seventeen made good recoveries, while eleven or eight and fifty-nine one-hundredths per cent. died. The usual death-rate was thirteen per cent. He says, "in strict justice to the test," five should not be counted for or against the treatment. This would leave one hundred and twenty-three cases treated, with a mortality of four and eighty-seven one-hundredths per cent., which will be conceded to be an unusually low rate. Under the old treatment the cases usually ran six weeks. Under the acetozone treatment the average was 28 days. There was less odor than before of the stools, and less odor in the wards. The stupor and delirium were much less, tympanites less frequent, and the diarrhea was checked. The temperature was reduced and the nurses commented on their lightened tasks when caring for typhoid cases under the acetozone treatment. Then he makes this significant statement: "From the information gained in watching this series of one hundred and twenty-eight cases of typhoid fever, I believe that where cases can be seen during the first week of the illness and given large amounts of acetozone solution regularly and often, assisted by a gentle laxative, the temperature will return to normal in from ten to twelve days." Dr. Flavel Woods and Dr. M. C. Thrust treated fifty-three cases of typhoid fever in the Presbyterian Hospital of Philadelphia during December, 1902, and January, 1903, without any deaths. They say relapses are infrequent when the remedy is given until convalescence is fully established. Dr. J. A. Art and Dr. E. Lackner, of Rush Medical College, treated forty cases. They publish a full report in the *Therapeutic Gazette*, at the end of which they make these statements: "Stupor and tympanites were almost entirely absent in our series of cases. The typhoid fetor of the stools was markedly diminished," etc. May

I add my mite to the greater mountain of experience already given to the medical world from which to formulate a more correct system of medicine? Almost a score of years ago I was attracted by the brilliancy of Rudolph Virchow, in cellular pathology, and Bouchard in autointoxication. I began to study, or rather to read, on these lines of thought until I was able to formulate a plan of treatment for typhoid fever, which has proved successful in fifty odd cases.

I begin the treatment by giving calomel in two-grain doses until I have given three doses. This I work off with oil or salts. I do not hesitate to give calomel at any time if it is indicated. I begin at the first indication of typhoid fever with the charcoal and sulpho-carbolate of soda treatment, giving two tablets every two hours, all the time until convalescence is complete. Each tablet contains charcoal gr. v, and sulpho-carbolate of soda gr. II. From one to two ounces of olive oil is given every night, and the colon flushed out every morning. If the kidneys become sluggish the patient is given an injection of normal salt solution per rectum. This has a splendid effect in stimulating the kidneys. Strychnine is given as soon as there is the first intimation of weakness. Milk diet is used all the time; also whiskey, plain or in egg-nog. Have not had a relapse or death, and there has been scarcely any fetor in the stools. The pinched and cloudy expression soon passes away, and the patient becomes cheerful. Osler had under his care in the Johns Hopkins Hospital eight hundred and twenty-nine cases, of which eighty-six, or ten per cent. had relapses; sixty-three, or seven and five-tenths of them, died. Dr. H. G. McCormick treated four hundred and eight cases, of which fifty-eight cases had relapses, and twenty-two, or five and four-tenths per cent., died. In sixteen of the leading hospitals the death rate was thirteen per cent. Do you ask me why I use the oil and the charcoal and sulpho-carbolate of soda? Dr. Paget, of Western Australia, regards olive oil as one of the most positive remedies in typhoid fever. Bouchard has demonstrated that charcoal given to typhoid fever patients reduces the poison in the discharges three-fourths. If you ask me how it does it, I must answer that I do not know. You all know that the sulpho-carbolates have proven themselves to be good intestinal antiseptics. To treat fifty cases with a certain line of treatment with no relapses and no deaths, gives me some encouragement to pursue the treatment still further. —*Pa. Med. Journal.*

